# Composite Polylactic-Methacrylic Acid Copolymer Nanoparticles for the Delivery of Methotrexate

**DOI:** 10.1155/2012/579629

**Published:** 2012-07-05

**Authors:** Bongani Sibeko, Yahya E. Choonara, Lisa C. du Toit, Girish Modi, Dinesh Naidoo, Riaz A. Khan, Pradeep Kumar, Valence M. K. Ndesendo, Sunny E. Iyuke, Viness Pillay

**Affiliations:** ^1^Department of Pharmacy and Pharmacology, Faculty of Health Sciences, University of the Witwatersrand, 7 York Road, Parktown, Johannesburg 2193, South Africa; ^2^Department of Neurology, Faculty of Health Sciences, University of the Witwatersrand, 7 York Road, Parktown, Johannesburg 2193, South Africa; ^3^Department of Neurosurgery, Faculty of Health Sciences, University of the Witwatersrand, 7 York Road, Parktown, Johannesburg 2193, South Africa; ^4^Department of Medicinal Chemistry, College of Pharmacy, Qassim University, Buraidah, Al-Qassim 51452, Saudi Arabia; ^5^School of Pharmacy and Pharmaceutical Sciences, St. John's University of Tanzania, Dodoma, Tanzania; ^6^School of Chemical and Metallurgical Engineering, University of the Witwatersrand, Private Bag 3, Wits, Johannesburg 2050, South Africa

## Abstract

The purpose of this study was to develop poly(lactic acid)-methacrylic acid copolymeric nanoparticles with the potential to serve as nanocarrier systems for methotrexate (MTX) used in the chemotherapy of primary central nervous system lymphoma (PCNSL). Nanoparticles were prepared by a double emulsion solvent evaporation technique employing a 3-Factor Box-Behnken experimental design strategy. Analysis of particle size, absolute zeta potential, polydispersity (Pdl), morphology, drug-loading capacity (DLC), structural transitions through FTIR spectroscopy, and drug release kinetics was undertaken. Molecular modelling elucidated the mechanisms of the experimental findings. Nanoparticles with particle sizes ranging from 211.0 to 378.3 nm and a recovery range of 36.8–86.2 mg (Pdl ≤ 0.5) were synthesized. DLC values were initially low (12 ± 0.5%) but were finally optimized to 98 ± 0.3%. FTIR studies elucidated the comixing of MTX within the nanoparticles. An initial burst release (50% of MTX released in 24 hours) was obtained which was followed by a prolonged release phase of MTX over 84 hours. SEM images revealed near-spherical nanoparticles, while TEM micrographs revealed the presence of MTX within the nanoparticles. Stable nanoparticles were formed as corroborated by the chemometric modelling studies undertaken.

## 1. Introduction

Much research has shown that, for optimal drug action, the most efficient way is to deliver the drug to the desired site of action in the body while attempting to decrease or avoid the side effects at nontarget sites [1–3]. Various drug delivery systems such as liposomes [4], micelles [5], and polymer micro/nanoparticles [6] have thus far shown promise in controlled release and targeted drug delivery. To date, biocompatible and biodegradable polymeric nanoparticles are the most preferred candidates for designing drug delivery systems [7]. Polymer-based nanostructured drug delivery systems have had a significant impact on biomedical technology, greatly enhancing the efficacy of many existing drugs and enabling the construction of entirely new therapeutic modalities [8]. Nanoenabled drug delivery systems have also demonstrated the ability to protect and target therapeutic compounds to the site of action and reduce the toxicity or side effects [9]. Biodegradable polymeric nanoparticles, in particular, have attracted considerable attention due to their ability to target particular organs/tissues and as potential carriers of DNA, proteins, peptides, and genes [10, 11].

Unezawa and Eto [12] prepared site-specific mannose liposomes from *p*-aminophenyl-*α* mannoside which were able to cross the blood-brain barrier (BBB) via the glucose transporter to eventually reach the mouse brain. Fenart and coworkers [13] prepared 1,2-dipalmitoyl-sn-glycero-3-phosphatidylcholine coated maltodextrin nanoparticles which were able to cross an *in vitro* model of the BBB and suggested an interaction of the coating with the BBB choline transporter. The physicochemical properties of nanoparticles are therefore important parameters in determining the physiological functions and stability of drug-loaded nanoparticles. Various studies have shown how to control the fabrication parameters in order to modulate the physicochemical aspects of drug-loaded nanoparticles for the delivery of macromolecules such as genes and proteins [14–16].

Thus far, polymeric nanoparticles ranging in size from 10 to 1000 nm have been synthesized from various biodegradable polymers such as polylactic acid (PLA), polylactic-coglycolic acid, (PLGA), chitosan, and poly(alkylcyanoacrylate) (PACA) [17–19]. The particle size of nanoparticles is one of the most significant determinants of BBB, mucosal, and epithelial tissue uptake including intracellular trafficking [20]. The surface charge of nanoparticles is another important determinant in not only playing a key role in stability, mucoadhesiveness, and permeation enhancement of nanoparticles [21, 22], but also the ability of nanoparticles to escape from endolysosomes [23]. The subcellular and sub-micrometer size of nanoparticles makes it possible for them to penetrate deep into tissues through fine capillaries and cross the fenestration present in the epithelial lining. This allows efficient delivery of therapeutic agents to target sites in the body such as the BBB [14, 24, 25].

The polymers PLA and PLGA have been widely used to synthesize polymeric nanoparticles due to their biodegradability and biocompatibility properties [26, 27]. These polymeric nanoformulations can be administered by varying routes of administration such as ocular, intravenous, topical, or oral [28–34]. Conventionally, nanoparticles have been prepared mainly by dispersion of the preformed polymers or by polymerization of monomers [35–39]. However, formulation of nanostructures from biodegradable polymers still remains a challenge [2, 38]. A few methods that have been proposed for preparing such polymer nanoparticles include solvent evaporation, nanoprecipitation, crosslinking, and salting-out [40–42]. Drugloading into the nanoparticles may be achieved by incorporating the drug at the time of nanoparticle synthesis or by adsorbing drug onto the surface of the produced nanoparticles by incubation in the drug solution [27]. Couvreur and coworkers [35] studied the adsorption of dactinomycin and methotrexate (MTX) on the surface of poly(methylcyanoacrylate) and poly(ethylcyanoacrylate), and it was observed MTX bound to the nanoparticles to a lesser extent [31].

Therefore, the aim of this study was to improve the adsorption of MTX onto biodegradable polymeric nanoparticles by preparing MTX-loaded nanoparticles from a combination of PLA and methacrylic acid copolymer (MAA). The PLA-MAA formulation was extensively characterized and optimized for its stability and MTX releasing ability. The main focus was to improve the drugloading of MTX. Both molecular structural modeling and molecular mechanics simulations were used for predicting preferred molecular conformations of the MTX polymer complexes using force-field minimizations, and the modes of interaction were envisaged in relation to the increase in MTX-loading/encapsulation efficiency. Quantitation of the MTX-polymer interactions from FTIR spectroscopy was also performed. Furthermore, force-field-based intermolecular interaction energies and molecular attributes were computed to investigate the geometrical preferences of the MTX-polymer complexes formed.

## 2. Materials and Methods

### 2.1. Materials

Poly(DL-lactide) (PLA) (Resomer R203H) was purchased from Boehringer Ingelheim Pharma GmbH (Germany). Methacrylic acid copolymer (MAA; Eudragit S100) was purchased from Degussa, Rohm GmbH, Pharma Polymers (Germany). Poly(ethylene glycol) (PEG6000) was purchased from Merck (Schuchardt OHG, Hohenbrunn, Germany). Sodium hydroxide (NaOH), dimethyl sulphoxide (DMSO), isopropyl alcohol, and dichloromethane (DCM) were purchased from Rochelle Chemicals (Johannesburg, South Africa), and methotrexate (MTX) was purchased from Sigma Aldrich (St Louis, MO, USA). All other reagents used were of analytical grade and were used as purchased.

### 2.2. Preparation of the MTX-PLA/MAA-Loaded Nanoparticles

A 3-Factor Box-Behnken experimental design was constructed for generating various MTX-loaded nanoparticle formulations (Table 1). The nanoparticles were prepared by a double emulsion solvent evaporation technique. The internal aqueous phase *(W1)* was prepared by dissolving 5 mg of MTX in a 1 mL solution of 0.1 M NaOH. The organic phase *(O)* was prepared by codissolving the polymers PLA and MAA in a mixed solvent system comprising dichloromethane and isopropyl alcohol in a ratio of 1 : 1. The quantities of PLA and MAA employed were in accordance with the 15 experimental design formulations template shown in Table 1. The internal aqueous phase and the organic phase were homogenized at 12,000 rpm (Polytron, PT 2000, Kinematika, AG Littau, Switzerland) for 3 minutes at room temperature (25 ± 0.5°C) to form a primary emulsion *(W1/O)*. The quantity ratios between the internal and organic phases also varied as per the experimental design template (Table 1). The external aqueous phase *(W2),* was prepared by dissolving PEG6000 in an acidic buffer (pH 2.0) to form a 2.5% w/v polymer solution. The primary emulsion *(W1/O)* was added dropwise to the external aqueous phase *(W2) *and emulsification was continued for further 10 minutes using a homogenizer to form nanoparticles. The formed nanoemulsion was centrifuged (Nison Instrument (Shangai) Limited, Shangai, China) at 15,000 rpm for 10 minutes at 25°C to recover the nanoparticles. The nanoparticles were then washed twice with deionized water using a Buchner funnel system and thereafter lyophilized (Lanconco, Kansas City, MS, USA) for 24 hours to obtain a stable free-flowing powder.

### 2.3. Determination of Particle Size Distribution, Zeta Potential, and Polydispersity Index

Particle size was measured by firstly dispersing 2 mg of nanoparticles in deionized water. The nanoparticle suspension was then filtered through a 0.22 *μ*m filter (Millipore, Billerica, USA) to remove any polymer agglomerates. The size of the nanoparticles was measured by dynamic light scattering (DLS) on a Zetasizer NanoZS instrument (Malvern Instruments, Worcestershire, UK). For absolute zeta potential and the polydispersity index (PdI) determination, nanoparticle formulation samples were immersed in deionized water and agitated to facilitate nanoparticle dispersion. The absolute zeta potential and PdI were then determined using the ZetaSizer NanoZS instrument.

### 2.4. Determination of the MTX-Loading Capacity from the Optimized Nanoparticles

The optimized nanoparticles were prepared as described earlier, in which case MTX was added during the nanoparticle formulation process. However, MTX loading was extremely poor as gauged from preliminary studies. In order to enhance the MTX loading capacity, MTX was added after synthesizing the nanoparticles and was therefore adsorbed onto the surface of the nanoparticles. This was achieved by incubating the nanoparticles in a concentrated solution of MTX. Briefly, 10 mg of MTX was partially dissolved in 0.7 mL of 50% methanol containing 1% DMSO. Nanoparticles (80 mg) were then accurately weighed and added to the MTX solution. The resultant suspension was then placed in an oven maintained at 30°C for 24 hours. Thereafter, the nanoparticles were dried at room temperature (25°C) for 24 hours prior to determining the MTX loading capacity. The quantity of MTX incorporated within the formulations was determined by adding nanoparticles to 10mL phosphate buffer saline (PBS, pH 7.4) and centrifuging at 3,000 rpm for 1 hour. The supernatant was analyzed for MTX content by UV spectrophotometry (Cecil 3021 Spectrophotometer, Cecil Instruments, Cambridge, UK) at 307 nm. The drug-loading was expressed both as MTX-loading (%) and MTX-content (%w/w) employing (1). All tests were conducted in triplicate (*N* = 3)

(1) Drug-loading (%)   =mass of MTX in the nanoparticlesmass of MTX used in the formulation×100,MTX content (%w/w)   =mass of MTX in the nanoparticlesmass of nanoparticles recovered×100.

### 2.5. *In Vitro* Drug Release Studies

*In vitro* release of MTX from the nanoparticles was evaluated in phosphate-buffered saline (PBS, pH 7.4). Nanoparticles were added directly into the dissolution medium and placed in an orbital shaking incubator set at 20 rpm with the temperature maintained at 37°C. At specified times, 5 mL samples of the release media were withdrawn and analysed by UV spectrophotometry (Cecil 3021 Spectrophotometer, Cecil Instruments, Cambridge, UK) at 307 nm. After sampling, the media was replaced with drug-free buffer (PBS, pH 7.4) of equal volume in order to maintain sink conditions. It was reported that this method is not very sensitive for studying rapid release formulations but can only be used for the release of formulations having drug release times for >1 hour [23].

### 2.6. Spectroscopic Analysis of the Nanoparticles Molecular Structure

MTX-loaded and drug-free nanoparticle samples were scanned over a wavenumber range between 4000 cm^−1^ and 650 cm^−1^ using a Perkin Elmer Spectrum 100 Series FTIR spectroscope (PerkinElmer LAS Inc. Waltham, MA, USA). Samples were placed on diamond crystals and processed by a universal ATR polarization accessory for the FTIR spectrum series. 

### 2.7. Assessment of Nanoparticle Morphology and Surface Characteristics

#### 2.7.1. Scanning Electron Microscopy

The shape and surface morphology of the nanoparticles were studied by scanning electron microscope (SEM) (Joel JSM-840, Tokyo, Japan). Samples were mounted on aluminium stubs and were sputter coated with gold platinum. The sample assembly was placed in the microscope and analysed at an accelerating voltage of 20 kV at various magnifications.

#### 2.7.2. Transmission Electron Microscopy

Nanoparticle size and shape were also explored using transmission electron microscopy (TEM) (JEOL 1200 EX, 120 keV). Samples were prepared by placing a dispersion of nanoparticles in ethanol on a copper grid with a perforated carbon film, followed by evaporation and viewing at room temperature at various magnifications.

### 2.8. Thermal Characterization of the PLA-MAA Copolymer Nanoparticles

Thermal analysis was performed on the constituent polymeric PLA-MAA nanoparticles using a temperature-modulated differential scanning calorimeter (TMDSC) (Mettler Toledo, DSC1, STAR^e^ System, Swchwerzenback, Switzerland) to assess the thermal behavioral transitions. Transitions were determined in terms of the glass transition temperature (*T*_*g*_), measured as the reversible heat-flow due to changes in the magnitude of the *C*_*p*_-complex values (Δ*C*_*p*_: melting (*T*_*m*_) and crystallization (*T*_*c*_) temperature peaks which are consequences of irreversible and reversible heat-flow corresponding to the total heat-flow). The temperature calibration was accomplished with the melting transition of indium. The transitions of the individual polymers were compared with the transition of the composite MTX-PLA-MAA nanoparticles. Samples were weighed (5 mg) on perforated 40 *μ*L aluminum pans, crimped, and then ramped from −35°C to 230°C on TMDSC under a nitrogen atmosphere in order to diminish oxidation at a rate of 1°C/min.The instrument parameters used are shown in Table 2.

### 2.9. Molecular Modeling Simulation of the Mechanisms of Nanoparticle Formation

Molecular structural modeling was performed to deduce a hypothesized mechanism of nanoparticle formation and potential interpolymeric interaction during nanoparticle formation. Semiempirical molecular theories were used to generate predictions of the molecular structure of the polymers and compute various molecular attributes using ACD/I-Lab, V5.11 software (Advanced Chemistry Development Inc., Toronto, Canada, 2000) based on the inherent interfacial phenomena underlying the formation of the MTX-loaded nanoparticles that were prepared by the double emulsion solvent evaporation technique. Models and graphics based on the stepwise molecular mechanisms of nanoparticle formation and PLA-MAA transitions as envisioned by the chemical behavior and stability were generated on ACD/I-Lab, V5.11 (Add-on) software (Advanced Chemistry Development Inc., Toronto, Canada, 2000).

#### 2.9.1. Molecular Mechanics (MM) Computations

Molecular mechanics computations in vacuum were performed using HyperChem 8.0.8 Molecular Modeling software (Hypercube Inc., Gainesville, FL, USA) and ChemBio3D Ultra 11.0 (CambridgeSoft Corporation, Cambridge, UK). The decamer of PLA and monomer of MAA were generated from standard bond lengths and angles employing the polymer builder tool on ChemBio3D Ultra in their syndiotactic stereochemistry as 3D models, whereas the structure of MTX was built with natural bond angles. The models were initially energy minimized using the MM+ force field, and the resulting structures were energy minimized using the AMBER 3 (Assisted Model Building and Energy Refinements) force field. The conformer having the lowest energy was used to create the MTX polymer complexes. A complex of one molecule with another was assembled by parallel disposition, and the procedure of energy minimization was repeated to generate the final models comprising PLA-MTX and MAA-MTX. Full geometrical optimization was performed in vacuum employing the Polak-Ribiere Conjugate Gradient method until an RMS gradient of 0.001 kcal/mol was reached. Force field options in the AMBER 3 (with all H-atoms explicitly included) and MM+ (extended to incorporate nonbonded limits and restraints) methods were set as defaults. For molecular mechanics calculations in vacuum, the force fields were utilized with a distance-dependent dielectric constant scaled by a factor of 1. The 1–4 scale factors were electrostatic = 0.5 and van der Waals = 0.5. For solvated systems, force field options in the AMBER (with all hydrogen atoms explicitly included) and MM+ (extended to incorporate nonbonded cutoffs, restraints, and periodic boundary conditions) methods were the HyperChem 8.0.8 defaults.

## 3. Results and Discussion 

### 3.1. Preparation and Constrained Optimization of the PLA-MAA Nanoparticles

**Figure 1:**
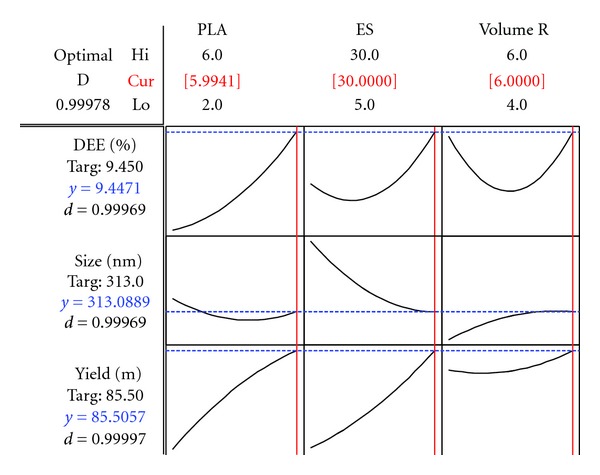
Desirability plots depicting the requisite variables for producing PLA/MAA nanoparticles with the desired targeted responses.

MTX-loaded nanoparticle formulations were obtained using the varying preparative variables stipulated by the 3-Factor Box-Behnken experimental design (Table 3). The choice of organic solvents used was mainly influenced by the solubility characteristics of PLA, MAA, and MTX. The double emulsion evaporation technique was adopted since it was superior to other incorporation methods in terms of encapsulating water soluble drugs. Upon adding the primary emulsion *(W1/O)* to the external aqueous phase *(W2)*, the mixture *(W1/O/W2)* became turbid indicating the spontaneous formation of nanoparticles. The counter outward diffusion of H_2_O and organic solvent into the emulsion nanoparticulate droplet, coupled with the gradual evaporation of the organic solvent, determined the *in situ* formation of the nanoparticles. The addition of PEG6000 in the external aqueous phase enhanced the stability of the formulations. Gradual addition of the primary emulsion into the external aqueous phase was crucial for preventing the formation of polymeric aggregates. In general, nanoparticle formation was satisfactory when the PLA-MAA solution was semidilute at intermediate phase volume ratios. This produced smaller particles with superior yields. It was also observed that formulation variables lying outside the selected limits (Table 4) resulted in nanoparticles with a high degree of aggregation. Based on the resultant responses obtained for the various formulations, the target particle size, MTX entrapment efficiency, and the yield were assigned for the optimization process. The requisite variables revealed optimized formulations with a particle size of 313 nm, yield of 85.5 mg, and a DEE value of 9.45% (Figure 1).

### 3.2. Effects of Formulation Variables on Nanoparticle Size and Zeta Potential

**Figure 2:**
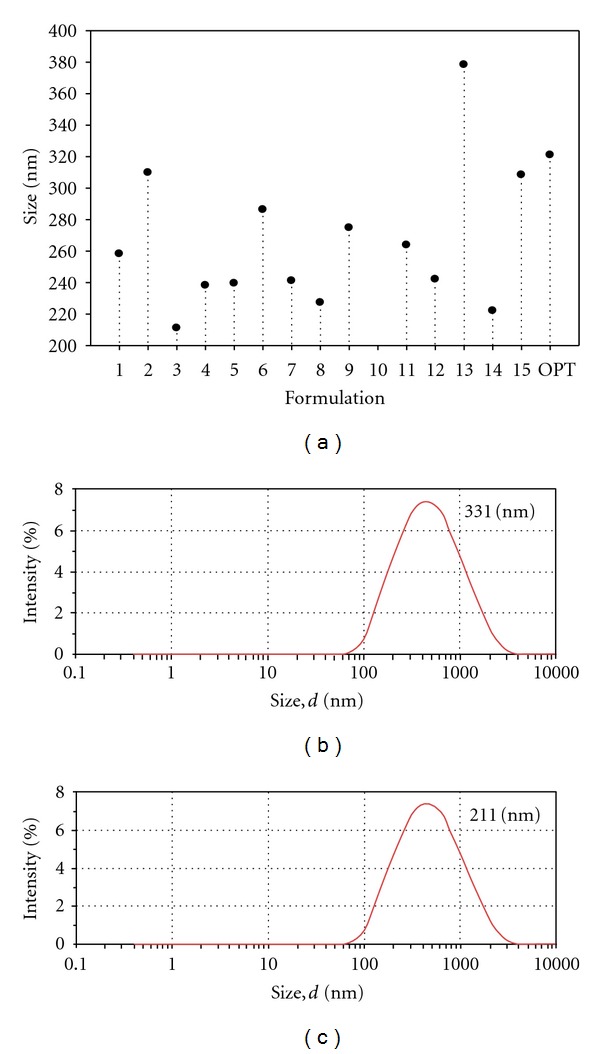
(a) Sizeplot depicting the sizes of different PLA/MAA nanoparticle formulations, (b) monomodal size distribution for the optimized PLA/MAA nanoparticle formulation, and (c) monomodal size distribution for the final PLA/MAA formulation.

**Figure 3:**
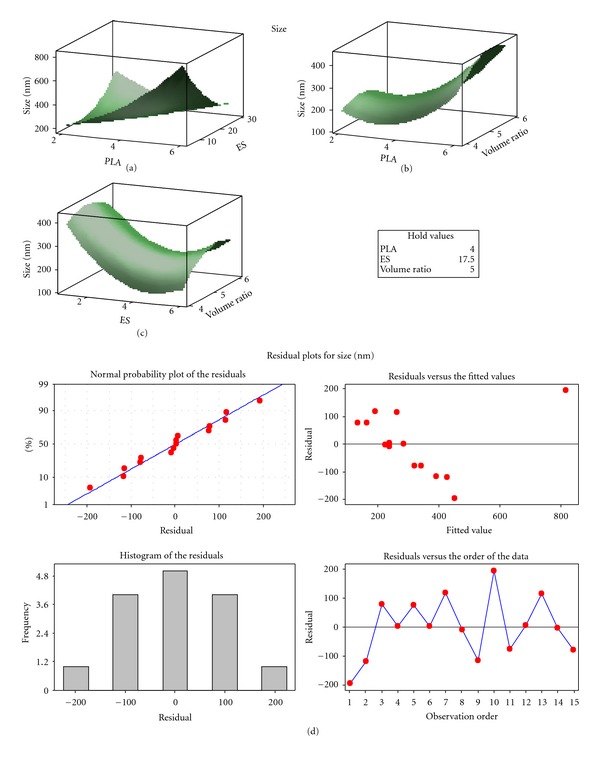
Residual plots for size distribution.

Nanoparticle size is an important parameter since it affects the MTX loading, drug release, and eventual site-specific delivery of MTX across the BBB. The nanoparticle sizes obtained from the experimental design formulations varied between 211.0 and 378.3 nm (Figure 2(a)). Formulations displayed polydispersity index (PdI) values of <0.5 which was an indication of a homogenous nanoparticle size distribution. The size distribution measurement indicated that the size of the optimized nanoparticles was 331 nm (Figure 2(b)). It was observed that the size of the optimized nanoparticles was reduced to 211 nm upon incubation in a concentrated MTX solution in an attempt to improve the MTX-loading capacity (Figure 2(c)). This effect was due to the insolubility of PLA and MAA in 50% methanol that resulted in nanoparticle size shrinkage. The reduction in size could have further been enhanced by the evaporation of the volatile solvent phase from the surface of the nanoparticles during the drying phase. Response surface plots showed that an increase in the quantity of PLA resulted in an increase in the nanoparticle size. However, an increase in the quantity of MAA had an antagonistic effect and resulted in a decrease in nanoparticle size. The phase volume ratio had no significant influence on the nanoparticle size. This was further evidenced by the residual plots of the particle size distribution (Figure 3). The absolute zeta potential values ranged from −0.048 mV to −1.070 mV. These zeta potential values indicate that the MTX-loaded PLA-MAA nanoparticles were fairly stabilized by electrostatic repulsion forces but may have the tendency to aggregate. For PCNSL therapeutic interventions, the optimized nanoparticles (211 nm) may be optimal for penetration into the neuronal-cellular architecture considering a pore size of 100–150 nm at the site of action [43]. The blood-brain barrier (BBB) penetration also needs to be considered as nanoparticles with a size >200 nm may not be able to penetrate through the BBB. Prospectively, other approaches such as (a) delivery of nanoparticles via the nasal route and (b) delivery in the form of a nano-enclatherated neuroerodible polymeric device can be used to deliver the nanoparticles in close vicinity to the lymphoma nodules.

### 3.3. Effect of Formulation Variables on the MTX-Loading Capacity within the PLA-MAA Nanoparticles

**Figure 4:**
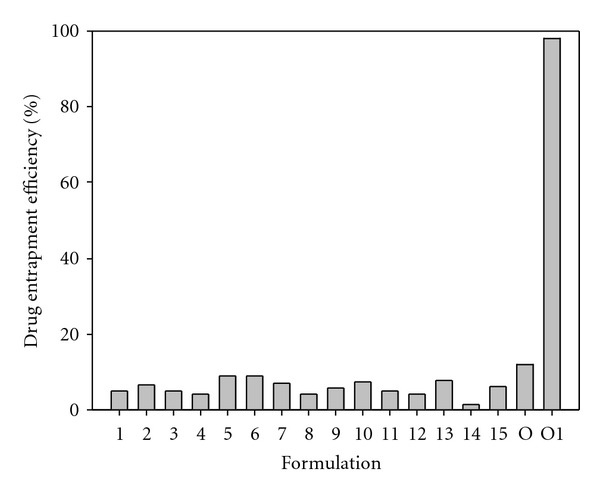
Barplot depicting differences in DEE within various PLA/MAA nanoparticle formulations.

**Figure 5:**
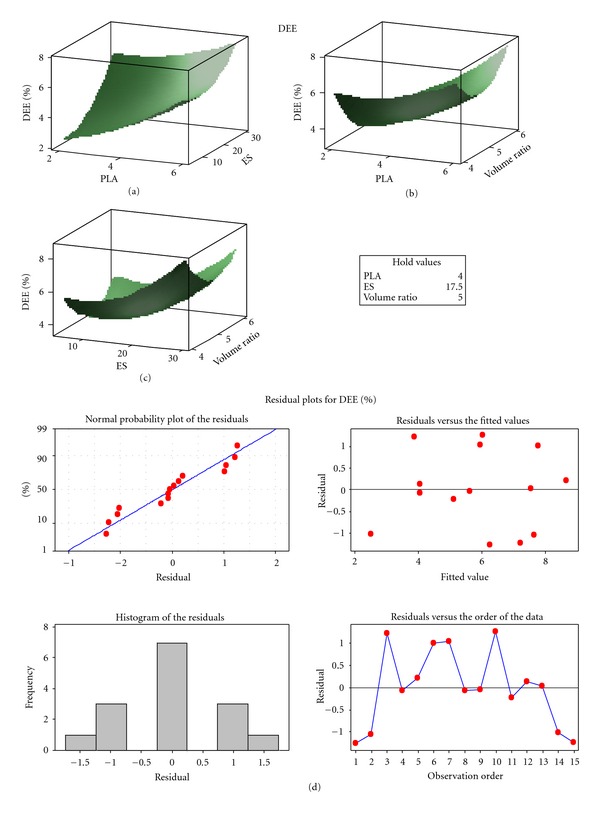
Residual plots for DEE.

Nanoparticle formulations from the experimental design showed poor MTX entrapment efficiency (Figure 4). Efforts to improve the DEE value by an optimization process proved futile with only 12% of MTX entrapped in the optimized nanoparticle formulation due to blending of PLA and MAA. This strategy did not lead to the formation of an amphiphilic polymer that was capable of entrapping MTX molecules during self-assembly with subsequent formation of nanoparticles with core-shell structure as described previously [37]. As a result, a high quantity of MTX molecules remained in solution during phase separation. Thus, this prompted investigation into an alternative approach to improve the MTX loading. Huafang and coworkers [44] have shown that drugs can be loaded onto the surface of particles and are more stable through surface adsorption on PLA nanoparticles. Therefore, optimized nanoparticle formulations were incubated into a concentrated MTX solution and allowed to cure in an oven at 30°C for 24 hours in an attempt to have the MTX adsorbed onto the PLA-MAA nanoparticle surface. This technique resulted in the MTX-loading capacity of the final formulation to significantly improved to 98%. In order for nanoprecipitation to occur, higher quantities of MAA and lower PLA were required to provide a dual polymer solution with suitable viscosity. Although the reason for poor MTX-loading could not be optimized any further, surface plots indicated that an increases in the quantities of PLA and MAA increased the DEE value. Intermediate phase volume ratios resulted in formulations with the lowest DEE value, while formulations with lower or higher phase volume ratios increased the DEE value. Residual plots for DEE are shown in Figure 5.

### 3.4. Effect of Formulation Variables on the PLA-MAA Nanoparticle Yield

**Figure 6:**
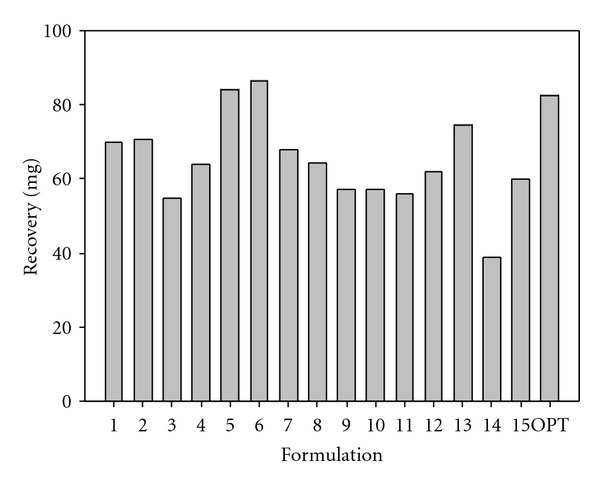
Barplot depicting differences in the yield within various PLA/MAA nanoparticle formulations.

**Figure 7:**
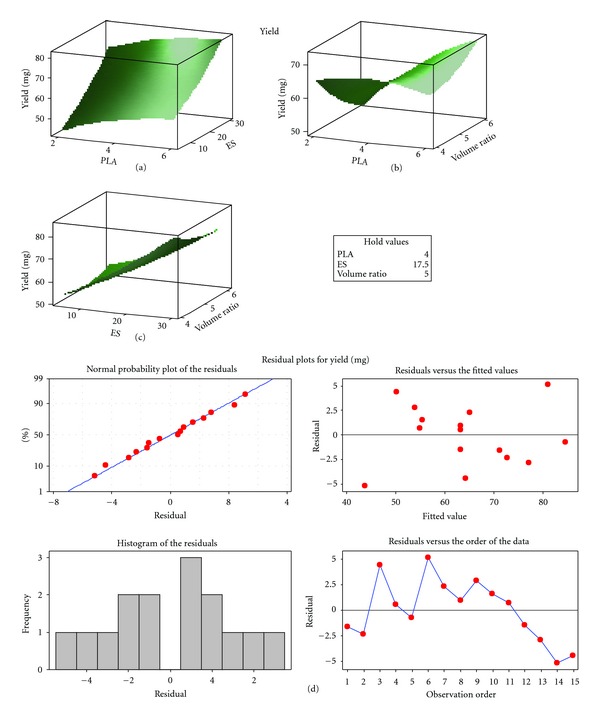
Residual plots for nanoparticle yield.

The yield of nanoparticles from the experimental design formulations was directly proportional to the quantity of PLA and MAA used. Yield values ranged between 36.8 and 86.2 mg (Figure 6). The yield for the optimized formulation was 82.4 mg and extremely close to the optimization target of 85.5 mg which was within the design space. Response surface plots showed that an increase in the quantity of PLA had a slight increase in the yield of nanoparticles. However, an increase in MAA resulted in a significant increase in the yield value, while the phase volume ratio had no significant influence on yield. Residual plots for the nanoparticle formulation yield are shown in Figure 7.

### 3.5. Molecular Structural Analysis of the PLA-MAA Nanoparticles

**Figure 8:**
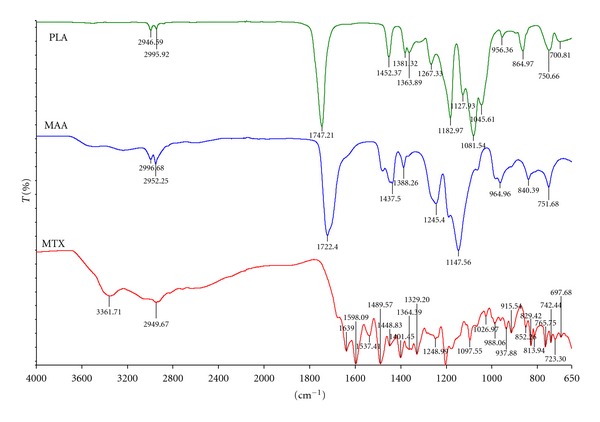
FTIR spectra of (a) methotrexate (MTX), (b) poly(DL-lactide) (PLA), and (c) methacrylic acid copolymer (1 : 2) (MAA).

**Figure 9:**
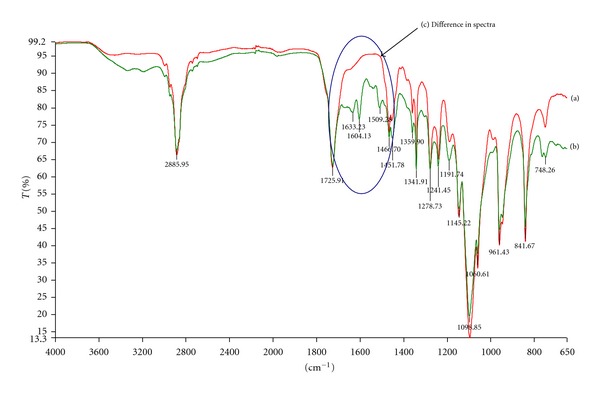
FTIR spectra of (a) drug-free PLA/MAA nanoparticles, (b) MTX-loaded PLA/MAA nanoparticles, and (c) highlighting the difference in the spectra.

The FTIR spectra of the drug-free and MTX-loaded optimized nanoparticle formulations corresponded to those of the native polymers (PLA and MAA) (Figures 8 and 9). This observation indicated that the polymers underwent minimal chemical change during processing. Therefore, it was expected that the nanoparticles would display chemical properties that were representative of the individual native polymers. Differences were noted in FTIR spectra between the drug-free and MTX-loaded nanoparticle formulations (Figure 8). The additional peaks that were observed in the MTX-loaded formulations were attributable to the presence of a 1,3 substituted compound (1509.36–1466.67 cm^−1^) and a phenyl amino compound (1633.22–1604.09 cm^−1^). This showed that MTX was adsorbed onto the nanoparticle surface either by weak H-bonds formed between the COO-groups of MTX and the OH-groups of MAA or by ionic bonds formed between the NH_2_ groups of MTX and the COO-groups present in PLA and MAA. MTX was dispersed in the PLA-MAA matrix in the microcrystalline form without polymorphic changes or transition into an amorphous form.

### 3.6. *In Vitro* Drug Release Studies

**Figure 10:**
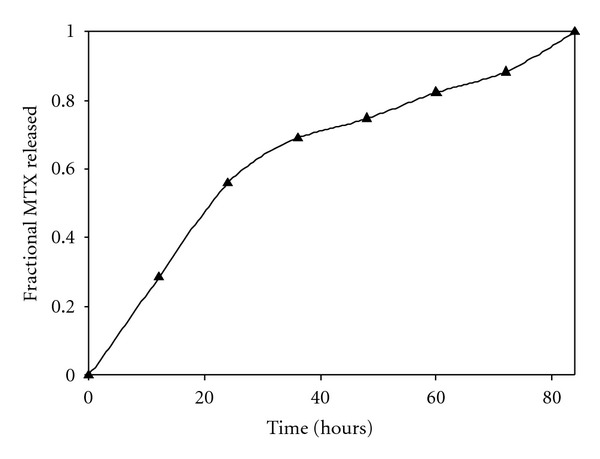
Release profile of MTX from an optimized PLA/MAA nanoparticle system with the highest drug incorporation efficiency.

*In vitro* release data of MTX indicated controlled release of MTX from the optimized nanoparticle formulation. As seen from the FTIR studies, PLA and MAA underwent minimal/no chemical transformation during nanoparticle synthesis. Therefore, the mechanism of MTX release was to an extent governed by the unique behavior of the constituent polymers in the release media. MAA is an ionic polymer that is gradually soluble in neutral to weakly alkaline media [39]. PLA is a pH-independent polymer that degrades extremely slowly in weakly alkaline media. MTX release occurred by diffusion of MTX molecules from the PLA-MAA matrix and followed a biphasic pattern (Figure 10). The first phase was attributed to the diffusion of MTX molecules that were weakly adsorbed onto the surface of the nanoparticles accounting for 50% of MTX released in 24 hours. Modulation of MTX release occurred during the second phase as a result of bond hydrolysis for which the subsequent release of MTX molecules dispersed within the inner matrix (Figure 10). The insolubility of MAA in the media prevented rapid matrix hydration and complete polymer chain relaxation. As a result, the PLA-MAA matrix maintained a tight interconnected networked structure and retarded the diffusion of MTX molecules. PLA imparted the nanoparticles with a certain degree of hydrophobicity, and its presence reduced the rate of matrix hydration by delaying the penetration of H_2_O molecules. The combined hydration, relaxation, and degradation kinetics of PLA and MAA in the dissolution media resulted in prolonged MTX release for over 84 hours (Figure 10). The *in vitro* drug release data demonstrated that the PLA-MAA nanoparticulate system can provide prolonged drug delivery (~80 hours) as compared to microparticles (12–25 hours) loaded with anticancer agents and prepared with different synthetic and natural polymer blends [45–47]. This prolonged rate of drug release allows the PLA-MAA system to be suitable for a nanoenclatherated neuroerodible polymeric device wherein the nanoparticles can be assembled as a layer-by-layer process and provide programmable drug release of the loaded nanostructure as well as bioactives for therapeutic management of PCNSL.

### 3.7. Morphological Characterization of the PLA-MAA Nanoparticles

**Figure 11:**
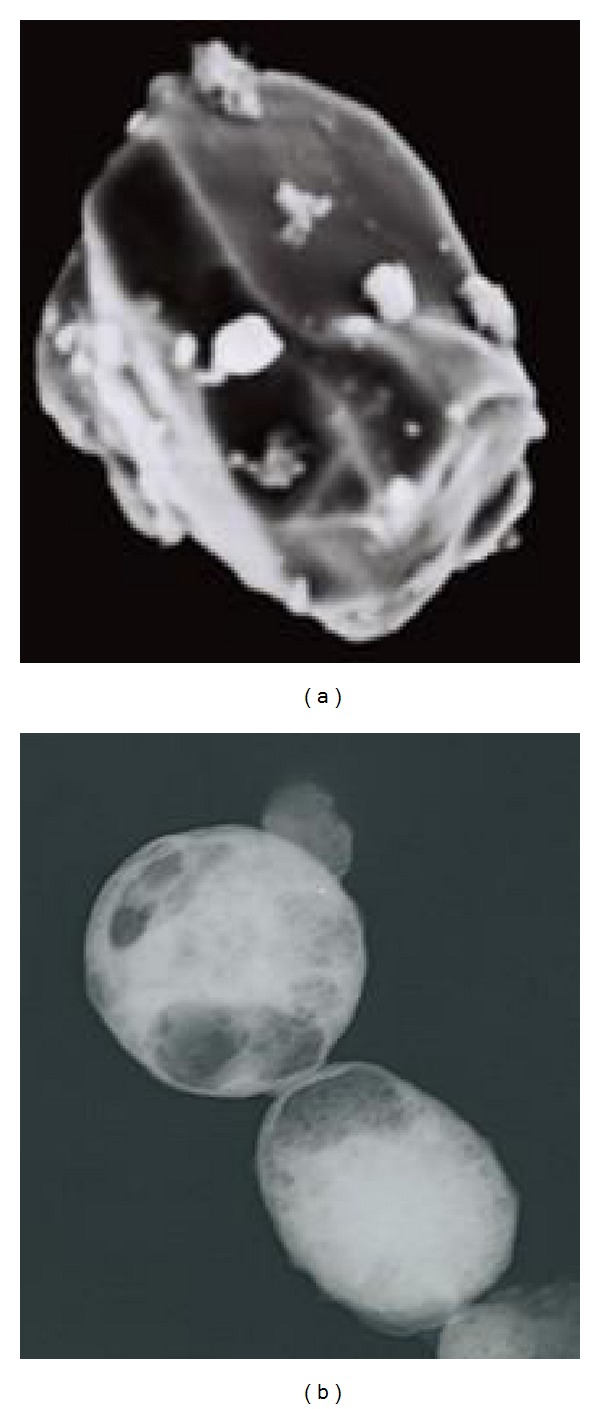
(a) SEM image showing the surface morphology of the optimized PLA/MAA nanoparticle formulation (x2500 magnification) and (b) TEM image of the optimized PLA/MAA nanoparticle formulation.

SEM micrographs revealed the presence of nanoparticles that were pseudospherical in shape. At higher magnification, the surface morphology revealed a collapsed PLA-MAA matrix as a result of the curing process in the presence of 50% methanol (Figure 11(a)). SEM also showed polymer aggregates that were adsorbed onto a smooth surface. TEM images confirmed the formation of matrix-type nanoparticles with a partially formed core-shell structure represented as clear areas in the micrograph (Figure 11(b)). 

### 3.8. Assessment of the Thermal Properties of the PLA-MAA Nanoparticles

**Figure 12:**
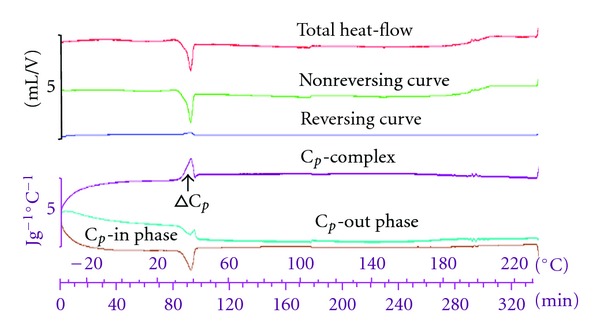
TMDSC profiles of PLA/MAA nanoparticles showing the endothermic and exothermic peaks generated from the reversible, nonreversible, total heat-flow curves, and the *C*_*p*_-complex, out-phase, and in-phase profiles that generated the reversible curves.

The thermal stability of the PLA-MAA nanoparticles was investigated by temperature modulated DSC (TMDSC) with a temperature range of −35–230°C. With TMDSC, the effects of baseline slope and curvature for the analysed samples became reduced thereby increasing the sensitivity of the system. Overlapping events such as molecular relaxation and glass transitions could be easily separated. With TMDSC, it was also possible to directly measure the *C*_*p*_. TMDSC utilizes sinusoidal temperature modulations with constant heating and cooling rates typified by short small amplitudes that were able to unveil and distinguish important hidden, overlapping thermal events within the MTX-loaded PLA-MAA nanoparticle matrix. The theoretical *T*_*g*_ for PLA is recorded between 50 and 80°C while the *T*_*m*_ value is between 173 and 178°C [48]. MAA has a theoretical *T*_*m*_ value of 100°C and a *T*_*g*_ that ranges between 85 and 165°C [49]. The signals for glass transition and for melting of the PLA-MAA composite appear in the reversing heat-flow signal of the TMDSC thermograms (Figure 12). TMDSC revealed a *T*_*g*_ value of 40°C (Figure 12) (i.e., lower than native PLA and MAA, thus indicating a shift to lower temperatures which is typical of PLA [50]). PLA is a relatively stiff and brittle polymer with low deformation at break [51]. It is also possible that the deconvolution of the total TMDSC signals for the PLA-MAA nanoparticles in the reversing and nonreversing events was lower than either of the two polymers. This is an indication that the melting component was predominantly reversing and resulted from the concurrent recrystallization and melting phenomena offsetting each other due to solid-to-solid phase transition during heating. The total heat-flow, reversing, nonreversing, *C*_*p*_ in-phase, and *C*_*p*_ out-phase curves showed a close association with the glass transition and relaxation phenomena of the amorphous PLA region. The exothermic and endothermic nonreversible events occurred simultaneously. This thermal behavior may have contributed to the controlled MTX release effect that was obtained since the permeability of the adsorbed MTX decreased as the polymers transitioned from an amorphous or glassy solid to a crystalline state. The controlled rate of MTX release would have most certainly been due to subsequent formation of a dense polymer matrix after blending PLA and MAA.

### 3.9. Molecular Mechanics Simulation of the Mechanisms of PLA-MAA Nanoparticle Formation

**Figure 13:**
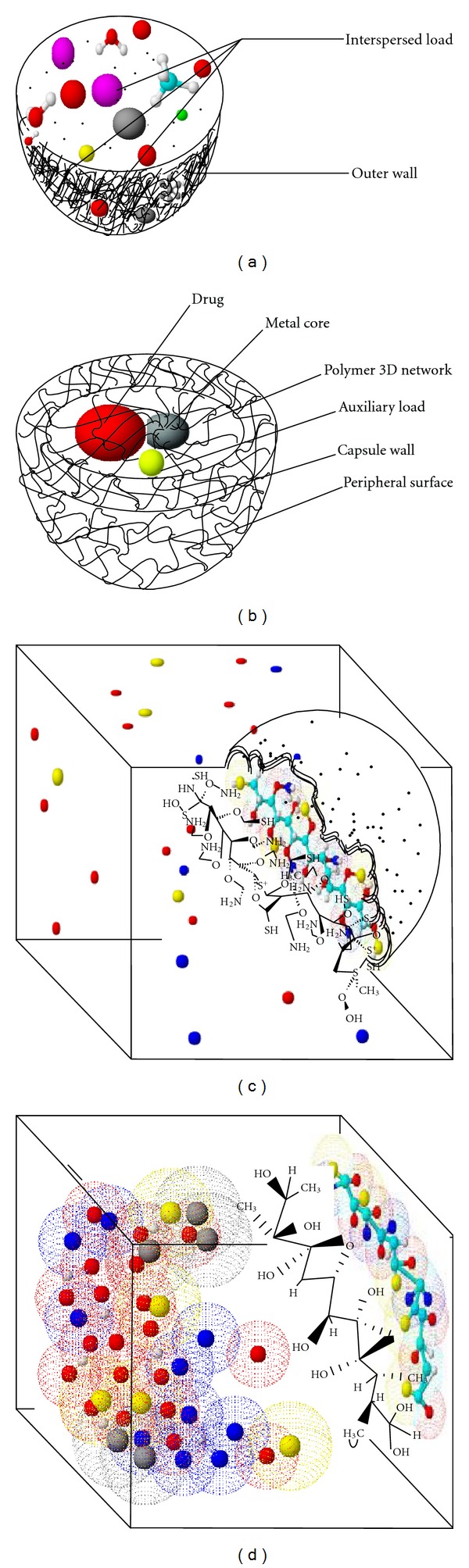
(a) Polymer MTX composite showing the interspersed load and the polymer outer wall, (b) MTX-PLA/MAA linkage depicted in a stereoorientation pattern, (c) a 3D model depicting the PLA-MAA surface embedding MTX, and (d) surrounding medium in an un-hydrated phase.

The mechanistic elucidation of PLA and MAA polymeric strand coalescence, chain interactions, and exchange of reactant and product molecules during dispersion in the nanoemulsification process have been molecularly simulated as shown in Figures 13(a)–13(d). When the coalesced PLA and MAA strands disperse within the crosslinking medium, excess reactant and newly transitioned sol-gel PLA and MAA molecules are redistributed into daughter strands. Nucleation of the PLA-MAA nanoparticle from the liquid-phase during the solvent evaporation process is depicted in Figure 13(a). Growth of the PLA-MAA nanoparticle by further sol-gel molecular interactions was mediated by coalescence exchange of polymeric strands and complete sphericalization. Coagulation of a multitude of sol-gel PLA and MAA molecules during coalescence of nucleated strands resulted in further particle size growth (Figures 13(b) and 13(c)). The ion balance, ion exchange, hydration, and interaction between hydrophilic sites in the PLA-MAA nanoparticle matrix and MTX were important parameters that facilitated the adsorption of MTX onto the PLA-MAA nanocomposite (Figure 13(d)).

**Figure 14:**
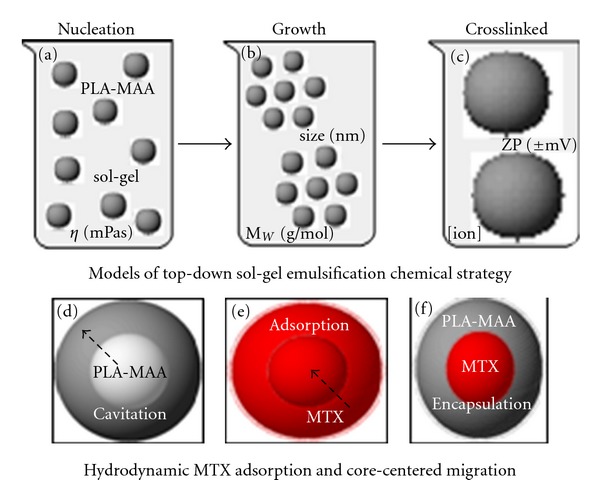
A computographic representation depicting (a) formation of uniform nanoparticle molecules (nucleation), (b) cluster or grouping of molecules (growth), (c) crosslinked nanoparticles, (d) ion fill with synthetic PLA/MAA cavitation, (e) MTX-PLA/MAA fill with MTX adsorption, and (f) heterogeneous fill depicting MTX loaded into the PLA/MAA composite.

Molecular models revealing the mechanisms of PLA-MAA nanoparticle formation employing the three top-down sol-gel emulsification chemical strategies demonstrated the simplicity, potential reproducibility, and stability of the nano-emulsions formed for PLA-MAA nanoparticle isolation (Figures 14(a)–14(f)). In hydrodynamic cavitation processing, nanoparticles are generated through the formation and release of gas bubbles within the sol-gel solution that is rapidly pressurized within a supercritical drying chamber and exposed to cavitational disturbances and high temperature heating [52]. The erupted hydrodynamic bubbles are responsible for nucleation, growth, and quenching of the nanoparticles with the particle size controlled by adjusting the pressure and the solution retention time in the cavitation chamber. This process is highly complex, and most polymers are susceptible to cavitation and high temperature, and this may result in premature degradation of the polymer. Thus, the top-down sol-gel double emulsion evaporation technique detailed in this study offers superior nanoparticle processing approaches (Figures 14(a)–14(f)).

### 3.10. Analysis of the Molecular Mechanics Computations

The monomer length for the polymer chain depicting molecular structures of PLA and MAA was determined on the basis of equivalent grid surface area (Table 5) enclosed by PLA and MAA so that the inherent stereoelectronic factors at the interaction site were perfectly optimized. The set of low-energy conformers that were in equilibrium with each other was identified and portrayed as the lowest energy conformational model.

**Figure 15:**
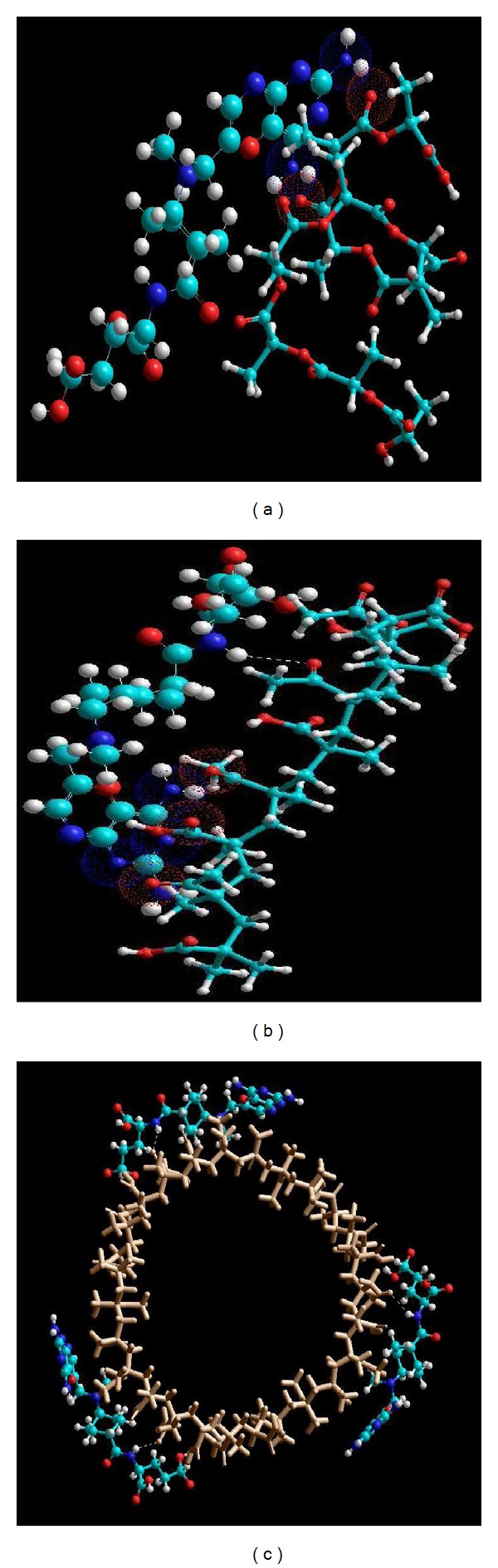
Energy-minimized geometrical preferences of the MTX-PLA-MAA complexes derived from molecular mechanics computations: (a) MAA-MTX, (b) PLA-MTX, and (c) depiction of adsorption of MTX on MAA nanoparticle (brown colored-tube rendered). MTX is rendered in spheres, and polymers are in ball/cylinder rendering. Color codes for elements are C (cyan), N (blue), O (red), and H (white).

The low-energy conformers of the PLA-MTX and MAA-MTX, that were in equilibrium with each other following molecular mechanics simulations, are depicted in Figure 15, and the possible component binding energies as well as the intrinsic molecular attributes to which they will be responsive are listed in Tables 5 and 6. Invariant factors common to mathematical description of binding energy and substituent characteristics have been ignored. It is evident from the energy values that the MAA-MTX complex was stabilized by a binding energy of 13.753 kcal/mol compared to 5.192 kcal/mol for PLA-MTX. These energy optimizations were supported mainly by the van der Waals interactions between MTX and the polymer molecule. Here, the MAA-MTX was stabilized with van der Waals forces by a magnitude of 14.488 kcal/mol compared to 6.954 kcal/mol for the PLA-MTX complex. This spatial preference of MAA over PLA is also depicted in the Figure 15 where upon deeper inspection revealed the close proximity of the MTX and MAA molecules. This was further confirmed by the surface-to-volume ratios (SVR) of the complexes with MAA-MTX having a lower SVR value than PLA-MTX (Table 5). The lower the SVR, the more stable the complex structure. Furthermore, a significant contribution was also provided by the strong H-bonding in MAA-MTX with a bond length of 2.6454 Å and the energy value exceeding nearly 50 times compared to PLA-MTX. These interactions involving the nonbonded attractive forces may induce dipoles in the complex where the binding energy transitions may be proportional to the polarizability of the substituents. These are in turn proportional to the molar refractivity values where the structure with the lower index of refraction is more stable. MAA-MTX was hence highly stabilized in comparison to the PLA-MTX with reference to refractivity (Table 6). 

These findings corroborated with the MTX-loading capacity that proved that MTX could be adsorbed onto the PLA-MAA nanoparticle surface. In addition, FTIR results were confirmed via the formation of amide linkages between the C-O*⋯*N-H groups of MTX and MAA/PLA, respectively. Although the PLA-MTX complex was less stable, the energy values, molecular attributes, and geometrical orientation were relatively comparable to the MAA-MTX complex. The MTX molecule displaying an energy-minimized extended conformation was superimposed onto a folded PLA molecule (Figure 15). Deeper inspection of the system revealed that the N_2_ atoms of MTX were in close interaction with the O_2_ atoms of the COO-groups of the PLA oligomer. These findings support the hypothesis of charge-dipole and dipole-dipole interactions between MTX and the polymers. This also explains the high efficacy of the PLA-MAA nanoparticles to adsorb MTX.

## 4. Conclusions 

Various formulations of PLA-MAA nanoparticles were successfully prepared by a double emulsion solvent evaporation technique using a randomized Box-Behnken statistical design template. The requisite variables required for producing an optimized MTX-loaded PLA-MAA nanoparticle formulation with the desirable response parameters were elucidated by desirability plots. The difference between the actual and desirable response values was minimal. Constrained optimization studies elucidated data on the interaction effects of the independent formulation variables such as the quantities of PLA and MAA as well as the phase volume ratio on the response parameters (particle size, MTX-loading capacity, and PLA-MAA nanoparticle yield). In general, the quantities of PLA and MAA had a significant influence on the response parameters, while variations in the phase volume ratio showed minimal influence. The MTX-loading capacity was significantly improved through MTX adsorption onto the PLA-MAA nanoparticle surface. SEM and TEM images confirmed the formation of matrix-type nanoparticles with small particle sizes and stable zeta potential values. Modulation and prolongation of MTX release from the PLA-MAA nanoparticles were achieved. The adsorption of MTX onto the nanoparticle surface as described in this study was stabilized by higher binding energies, van der Waals forces, shorter H-bond lengths, low surface-to-volume ratios, and low indices of refraction. Further studies are aimed at incorporating the synthesized nanoparticles within a neurodurable scaffold for delivery across the BBB.

## Figures and Tables

**Table 1:** Arrangement of the 3-factor Box-Behnken experimental design for PLA-MAA nanoparticle formulation.

Formulation number	Quantity of PLA (mg)	Quantity of MAA (mg)	Ratio W1: O (mL)
1	2	30.0	1: 5
2	6	17.5	1: 6
3	2	17.5	1: 6
4	4	17.5	1: 5
5	4	30.0	1: 4
6	6	30.0	1: 5
7	2	17.5	1: 4
8	4	17.5	1: 5
9	4	5.0	1: 4
10	6	5.0	1: 5
11	4	5.0	1: 6
12	4	17.5	1: 5
13	4	30.0	1: 6
14	2	5.0	1: 5
15	6	17.5	1: 4

**Table 2:** Temperature-modulated differential scanning calorimetry settings employed for thermal analysis of the PLA-MAA nanoparticles.

Segment type	Parameter setting
Sine phase^a^	
Start	−35^°^C
Heating rate	1^°^C/min
Amplitude	0.8^°^C
Period	0.8^°^C
Loop phase^b^	
To segment	1
Increment	0.8^°^C
End	230^°^C
Count	436

^a^Sinusoidal oscillations.

^b^Oscillation periods.

**Table 3:** Response data obtained for the 3-factor Box-Behnken experimental design PLA-MAA nanoparticle formulations.

Formulation number	Size (nm)	PdI value	DEE (%)	Yield (mg)
1	258.2	0.255	5.0	69.65
2	309.8	0.371	6.6	70.45
3	211.1	0.237	5.1	54.60
4	238.2	0.365	4.0	63.80
5	239.5	0.289	8.9	84.00
6	286.3	0.277	8.8	86.20
7	308.4	0.413	7.0	67.50
8	227.3	0.297	4.0	64.20
9	274.8	0.388	5.6	56.85
10	1012.0	0.971	7.3	57.10
11	263.8	0.197	4.9	55.70
12	242.1	0.354	4.2	61.80
13	378.3	0.250	7.6	74.40
14	222.1	0.682	1.5	38.60
15	241.1	0.281	6.0	59.90
Optimized 1	331.0	0.289	12	82.40
Optimized 2	211.0	0.284	98	82.40

**Table 4:** Formulation constraints employed for response optimization.

Variables	Limits
Quantity of PLA	2–6 mg
Quantity of MAA	5–30 mg
Ratio (*W*1/*O*)	1: 4–1: 6

**Table 5:** Computed molecular attributes of the complexes involving PLA, MAA, and MTX.

Structure	Molecular attributes				
	Surface area (grid)	Volume (cm^3^)	Surface-to-volume ratio	Refractivity	ΔRef^a^
PLA	922.53	1828.26	0.5046	156.96	—
MAA	987.05	1954.76	0.5049	195.59	—
MTX	726.43	1255.51	0.5786	114.60	—
PLA-MTX	1373.47	2855.10	0.4811	270.67	−0.89
MAA-MTX	1315.76	2874.97	0.4577	307.21	−2.98

^a^ΔRef = Ref_(Host.Guest)_− Ref_(Host)_− Ref_(Guest)_.

**Table 6:** Computed energy parameters (kcal/mol) of the complexes involving PLA, MAA, and MTX.

Structure	Energy (kcal/mol)				
	Total	ΔE_binding_^a^	vdW^b^	ΔE_vdw_^c^	H-bond
PLA	1.713	—	−8.776	—	−0.006
MAA	62.382	—	16.840	—	−0.010
MTX	9.457	—	4.845	—	0
PLA-MTX	5.978	−5.192	−10.885	−6.954	−0.009
MAA-MTX	58.086	−13.753	7.197	−14.488	−0.456

^a^ΔE_binding_ = E_(Host.Guest)_− E_(Host)_− E_(Guest)_.

^b^van der Waals contribution.

^c^ΔE_vdw_ = Vdw_(Host.Guest)_− VdW_(Host)_− VdW_(Guest)_.
